# A Review on Hierarchical Nanostructures for Electrochemical Sensors

**DOI:** 10.3390/s26010073

**Published:** 2025-12-22

**Authors:** Safia Dassallem, Khalid Nouneh, Yanpeng Xue, Domenica Tonelli, Abdelhafed Taleb

**Affiliations:** 1 Laboratory of Materials Physics and Subatomics LPMS, Faculty of Sciences, University Ibn Tofail, Kenitra 14000, Morocco; safia.dassallem@gmail.com; 2 National Center for Materials Service Safety, University of Science and Technology Beijing, Beijing 100083, China; xueyanpeng789@163.com; 3 Department of Industrial Chemistry “Toso Montanari”, University di Bologna, Via Piero Gobetti 85, 40129 Bologna, Italy; domenica.tonelli@unibo.it; 4 Sorbonne University, 4 Place Jussieu, 75231 Paris, France; 5 Laboratoire Interfaces Traitements Organisation et DYnamique des Systèmes, CNRS UMR-7086, Paris Cité Université, 75013 Paris, France

**Keywords:** hierarchical nanostructures, electrochemical sensors, electroanalysis

## Abstract

In recent years, researchers have significantly increased their exploration of nanomaterials, primarily due to their exceptional and distinctive electrochemical properties. Hierarchical nanostructured materials have become a prevalent component in electrochemical sensors owing to their numerous advantages, including abundant open diffusion channels, diverse junction interfaces, and a highly exposed surface area. This review provides a comprehensive overview of the potential of hierarchical nanomaterials as electrode modifiers, highlighting their capacity to enhance device performance. The introduction section sets the context by addressing the challenges and recent advancements in the field of hierarchical nanomaterials, emphasizing their promise for electrochemical sensor applications, and outlining the diverse research directions that are currently being explored. In the following section, a range of strategies and techniques for synthesizing hierarchical nanomaterials are outlined, with an emphasis on the impact of various parameters on their properties. Subsequently, the characteristics and performance of diverse hierarchical nanomaterials as electrode modifiers for electrochemical sensor applications are examined. Ultimately, the primary aspects and challenges of hierarchical nanomaterials in the domain of electroanalysis are reported, followed by a discussion of their future development.

## 1. Introduction

Hierarchical materials can be defined as solid structures with different levels of substructure scale [1]. Nature has pioneered the fabrication of these materials, which scientists are attempting to reproduce using a variety of approaches. In some cases, parts of the whole surface of hierarchical materials can be repeated on a smaller scale; in such cases, the surface can be fractal. This class of materials offers new opportunities to control the properties of materials, optimize existing ones, or give rise to new functionalities. This enables the creation of customized materials and the emergence of an extensive range of new applications [2,3].

The field of nanomaterials has recently attracted significant interest from the scientific community, primarily due to the unique properties of these materials and the extensive range of potential applications they offer across various sectors. Within the domain of nanotechnology, the development of nanomaterials with novel architectures has emerged as a pivotal concern, both from a fundamental perspective and in terms of practical applications, including sensors and biosensors, catalysts, and energy conversion and storage [4,5,6,7]. In recent years, a variety of one-dimensional, two-dimensional, and three-dimensional nanostructured materials have demonstrated novel properties, resulting in technological advancements across multiple disciplines [8]. Hierarchical nanomaterials belong to this category of materials and are composed of smaller constituent units, termed nano-units, including nanoparticles, nanorods, nanowires, nanotubes, and nanoplatelets. These materials exhibit extraordinary properties, rendering them promising candidates for various applications in the field of highly active catalysts, ultrasensitive sensors, and host materials with high adsorption capacity [9,10,11].

The presence of toxic substances in the environment represents a significant threat to the integrity of the natural ecosystem and the well-being of human populations. In order to mitigate the associated risks, it is imperative to implement effective early detection procedures and adhere to the principles of good practice. Such measures are essential in preventing the deleterious consequences of these substances on public health and the ecosystem as a whole [12].

In this context, the combination of nanomaterials and electrochemical techniques has enabled the development of highly effective sensors capable of detecting toxic substances at trace levels. Indeed, nanomaterials have facilitated the fabrication of electrodes with controlled properties, which has in turn enabled a more profound understanding of electrochemical kinetics at even smaller scales. Additionally, the surface roughness and/or porosity present at the nanoscale level have enabled the electrode surface to expose a substantial specific surface area and, consequently, a considerable number of electroactive sites, thereby enhancing adsorption and chemical reactions at the electrode surface [4,13]. The 3D nanostructuring of sensors to maximize the exposure of active sites is an aspect that has not been widely addressed in the current state of the art, although it is a promising route to produce new photocatalytic materials with higher levels of performance. The nanoscale of materials also facilitates mass, heat, and charge transfer, as well as all the concomitant chemical reactions [14]. Furthermore, sensors with a micro/nano hierarchical structure are evolving towards miniaturization, digitalization, intelligence, and systematization, and consequently, towards the increased performance needed for better monitoring of the presence of toxic substances in the environment and the human body.

Electrochemical analytical techniques are distinguished by their simplicity of implementation, high sensitivity, and proven effectiveness in detecting toxic pollutants in the environment and in biological media [15,16]. The development of electrochemical sensors and biosensor platforms is of key importance in preventing risks from toxic substances [17]. The requirement for efficient and cost-effective detection devices for continuous monitoring of toxicological hazards is a major concern of governments worldwide for environmental and food safety, as well as for biological and biomedical applications [18].

The development of nanomaterials to improve the performance of electrodes is of growing interest in the materials field, due to their major role in current issues such as energy storage and conversion, environmental protection, and healthcare.

The modification of electrodes with nanomaterials provides several advantages, including (1) surface activation and catalysis, (2) biocompatibility through appropriate functionalization or conjugation of nanomaterials, (3) accessibility, and (4) a broad spectrum of chemical reactions [14]. Hierarchical nanomaterials with different structural scales offer other remarkable advantages at the electrode surface, such as the formation of abundant open diffusion channels, diverse junction interfaces, and the exposure of a large surface area of active sites [19].

Several authors have published a review on the use of hierarchical nanomaterials in electrochemical devices, including batteries, photoelectrochemical cells, fuel cells, and sensors [4,5,6,7,13]. Wang et al. have reviewed the state of the art of hierarchical nanomaterials in the construction of high-performance supercapacitors [6]. Over the past decade, there has been significant research and development in the field of hierarchical nanomaterials concerning electrochemical sensors, leading to the development of high-performance electrochemical devices. Given the continuous evolution of the field of sensors, there is an urgent need to review the new hierarchical structures used in electrochemical sensors. Such a review would analyze the advantages and disadvantages of their synthesis methods and discuss the performance of the hierarchical nanomaterial-based sensors.

However, despite the availability of several reviews, a careful examination of the existing literature reveals important gaps that remain unaddressed. First, most previously published reviews focus broadly on nanomaterials for energy storage or general electrochemical applications, but they do not specifically dissect the role of hierarchical architectures such as multilevel core-shell structures, hollow frameworks, or 3D interconnected networks in electrochemical sensing. Second, existing reviews tend to list synthesis methods without providing a critical comparison of their scalability, structural controllability, reproducibility, and suitability for constructing complex hierarchical morphologies. Third, it’s hard to develop a systematic and quantitative explanation of how hierarchical organization, interfacial characteristics, and electrochemical performance (sensitivity, selectivity, LOD, and stability) are all related. Finally, no comprehensive investigation has yet integrated these components to provide a unified perspective on how hierarchical design principles boost sensing capabilities across diverse analyte classes. This paper aims to address these deficiencies by providing a focused and contemporary analysis of hierarchical nanostructures specifically designed for electrochemical sensing.

**Figure 1:**
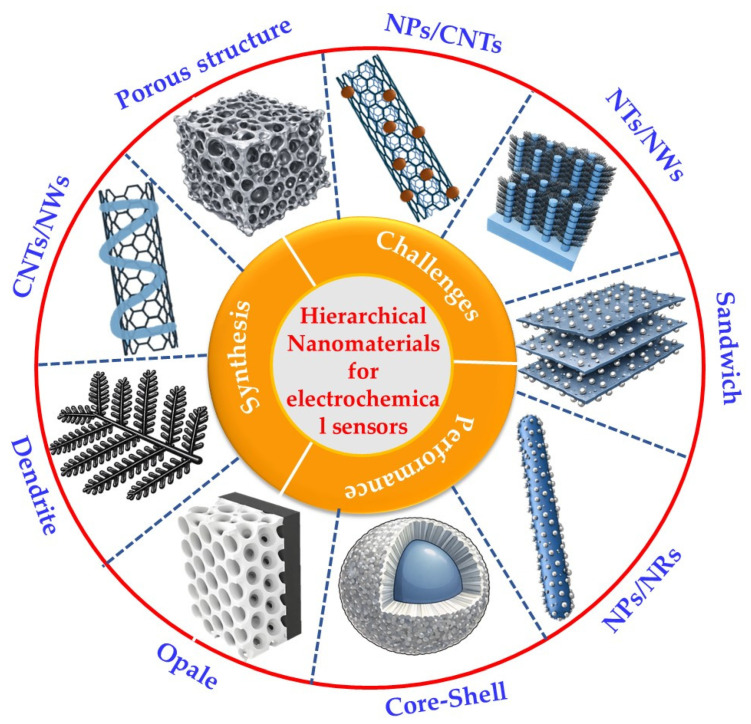
The scheme shows the objectives of the review and different hierarchical materials used for electrochemical sensors.

The present review is structured in three sections. The first section presents various strategies and techniques for synthesizing hierarchical nanomaterials, as illustrated in Figure 1, including an examination of the effect of different parameters on their properties. The second section reviews the characteristics and applications of hierarchical nanomaterials for electrochemical sensors. The third and final section discusses the main challenges of using hierarchical nanomaterials in electrochemical sensors and anticipates their future development.

## 2. Synthesis of Hierarchical Nanomaterials

**Figure 2:**
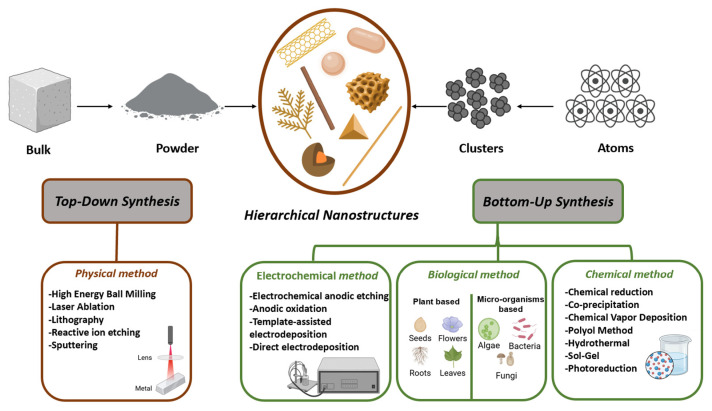
Top-down and bottom-up approaches for hierarchical nanostructures synthesis.

The synthesis of hierarchical nanomaterials is achieved through two distinct approaches: the “bottom-up” and the “top-down” methods (Figure 2). The bottom-up approach involves the preparation of nanomaterials atom by atom, molecule by molecule, or aggregate by aggregate. The assembly and arrangement of atoms, molecules, or aggregates are conducted in a precise and controlled manner, enabling the development of functional and structured materials. In contrast, the “top-down” approach involves the division of a material from a macroscopic state until it attains nanometric dimensions [20]. The primary techniques employed in this approach include high-energy milling, as well as optical and electronic lithography. The synthesis of hierarchical nanostructures can be categorized into three distinct approaches: direct growth on a substrate or in solution, assembly of nano-units (e.g., nanoparticles, nano-rods, nanowires, nanotubes, and nano-webs), and templated synthesis using alumina matrices.

A variety of experimental methods belonging to the above-mentioned families have been utilized in the fabrication of hierarchical nanostructure-based electrochemical sensors. These methods include conventional chemical synthesis, electrochemical methods, physical methods, and biological methods [21,22,23]. In general, the construction of hierarchical nanomaterials may require the combination of one or more synthesis methods. The chemical reduction technique is one of the most frequently used methods for the synthesis of nanostructured materials, and its reaction parameters, such as temperature, pH, synthesis time, and concentration of reagents, have a considerable impact on the structure and size of the resulting nanomaterials [24]. Electrochemical techniques have many advantages over chemical reduction methods, such as speed and ease of execution, low cost, mild temperature conditions, high yield, and environmental friendliness [25]. Moreover, electrochemical technologies are also suitable for the large-scale synthesis of nanostructured materials. However, it should be noted that the surface of the electrodes has the potential to restrict the synthesis yield of nanomaterials. The employment of ultrasound-assisted techniques has become a widespread practice for the synthesis of novel nanomaterials, as the physical and chemical environment engenders accelerated synthesis processes and diminutive crystal sizes. In comparison with chemical approaches, ultrasonic chemical reactions have been shown to be less harmful to the environment. They are associated with reduced costs, enhanced reaction rates, and increased yields, as well as homogeneous morphology and particle size dispersion of the final products [26]. Furthermore, laser-assisted approaches have been demonstrated to possess the capability to adjust reaction parameters, such as laser power, wavelength, and reaction time, in addition to controlling material characteristics, including shape, size, and crystallinity. A further advantage of the laser-assisted method is that it does not require the use of stabilizing modifiers or surfactants [27]. The subsequent discussion will focus on three synthesis approaches in depth.

Significant differences exist between chemical and physical synthesis approaches. Chemical methods generally offer precise control over composition, doping, and surface chemistry, which facilitates the formation of complex hierarchical structures through controlled nucleation and growth. These techniques, however, often require cleaning steps involving detergents or reducing agents. In contrast, laser ablation, ultrasonic fragmentation, and plasma-assisted physical methods enhance sample cleanliness, eliminate chemical residues, and accelerate fabrication. Nonetheless, they may result in metastable phases or defective structures, and without proper calibration, they can limit control over crystallinity, particle size distribution, and hierarchical organization. Ultimately, the choice between chemical and physical methods depends on whether the priority is achieving exceptionally clean, residue-free surfaces (physical routes) or obtaining fine control over structural features (chemical routes).

Biosynthesis-based strategies have recently emerged as compelling alternatives to conventional physical and chemical approaches for producing hierarchical nanostructures, particularly in applications emphasizing green chemistry, biocompatibility, and non-toxic processes. Biological synthesis routes employing plant extracts, microorganisms, enzymes, or biomolecules offer several advantages, including mild operating conditions, the elimination of hazardous reducing agents, intrinsic capping and stabilizing effects provided by natural metabolites, and relatively low environmental and economic costs. These features make biosynthetic pathways especially attractive for the development of electrochemical sensors used in biomedical and environmental monitoring. However, these methods also present notable limitations, particularly in terms of batch-to-batch reproducibility, slower reaction kinetics, and reduced control over the fine-tuning of hierarchical architectures such as particle size distribution, morphology, and crystallinity compared with conventional chemical or physical techniques. The following discussion provides an in-depth review of these three synthesis approaches.

**Figure 3:**
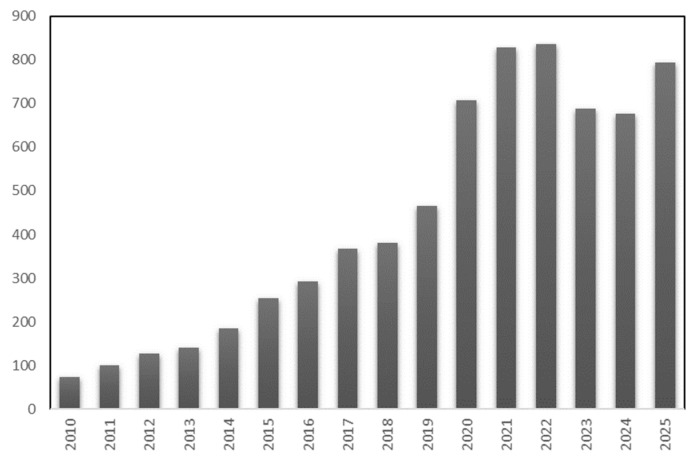
Number of Publications on Hierarchical Nanostructures for Electrochemical Detection (2010–2025).

The synthesis principles outlined above have established the foundation for a rapidly expanding field of research. To contextualize this development, the number of annual publications on hierarchical nanoparticles for electrochemical detection between 2010 and 2025 was analyzed (Figure 3). The histogram reveals a clear and steady upward trend, indicating that research in this area is continuously growing. From 2010 to 2016, a gradual increase is observed, reflecting the initial development of hierarchical architectures and their integration into electrochemical detection systems.

After 2017, the growth became more pronounced. In parallel, more advanced bottom-up and hybrid assembly techniques emerged, enabling the fabrication of multi-scale functional nanostructures that enhance electron mobility and improve analyte accessibility. The period from 2020 to 2022 records the highest number of publications, partly due to the COVID-19 pandemic, which heightened interest in hierarchical design concepts and boosted scientific productivity.

Although a slight decline is observed in 2023 and 2024, the overall publication level remains significantly higher than in the previous decade, confirming the sustained strength and continuity of research interest. Projections for 2025 reinforce this trend, suggesting that hierarchical nanostructures continue to represent a rapidly evolving field with expanding applicability in advanced electrochemical detection.

### 2.1. Synthetic Approach Based on Direct Growth

A variety of conventional chemical syntheses, including the hydrothermal method, chemical reduction, redox method, galvanic displacement reaction, sol–gel method, and chemical vapor deposition, are frequently employed in the synthesis of nanostructured materials. These methods are generally considered to be straightforward and uncomplicated. Moreover, the enhanced control over material synthesis parameters enables the regulation of their properties, including morphology, composition, and structure. This, in turn, can facilitate the conception of new hierarchical material architectures. Among the methods frequently used to control the structure and morphology of materials, the electrochemical deposition technique is a simple, low-cost, and efficient method compared to other physical methods such as evaporation, sputtering, and molecular beam epitaxy. In addition to the opportunity of working in solution without the use of costly vacuum technologies, the electrochemical deposition process offers the possibility of using the potential as a parameter for fine-tuning the deposition conditions and, consequently, the architecture and morphology of the deposited films. In physical methods, the control of the shape, size, and density of the deposits is mainly achieved by the deposition rate and surface diffusion [28], whereas, in the electrochemical process, numerous parameters such as potentials, current, additives, solvent, pH, and temperature must be taken into consideration. Furthermore, the morphology of the electrodeposited materials is determined by the interplay between the nucleation and growth processes that occur during electro-crystallization [29,30,31]. The equilibrium between these processes is governed by surface diffusion and deposition flux. Furthermore, the efficacy of spikes formed during electrodeposition to attract ions from the electrolyte, owing to the enhancement of the electric field in their vicinity, and the presence of additives (surfactants) in the electrolyte [32,33], can exert a substantial influence on the material morphology if metals are electrodeposited.

**Figure 4:**
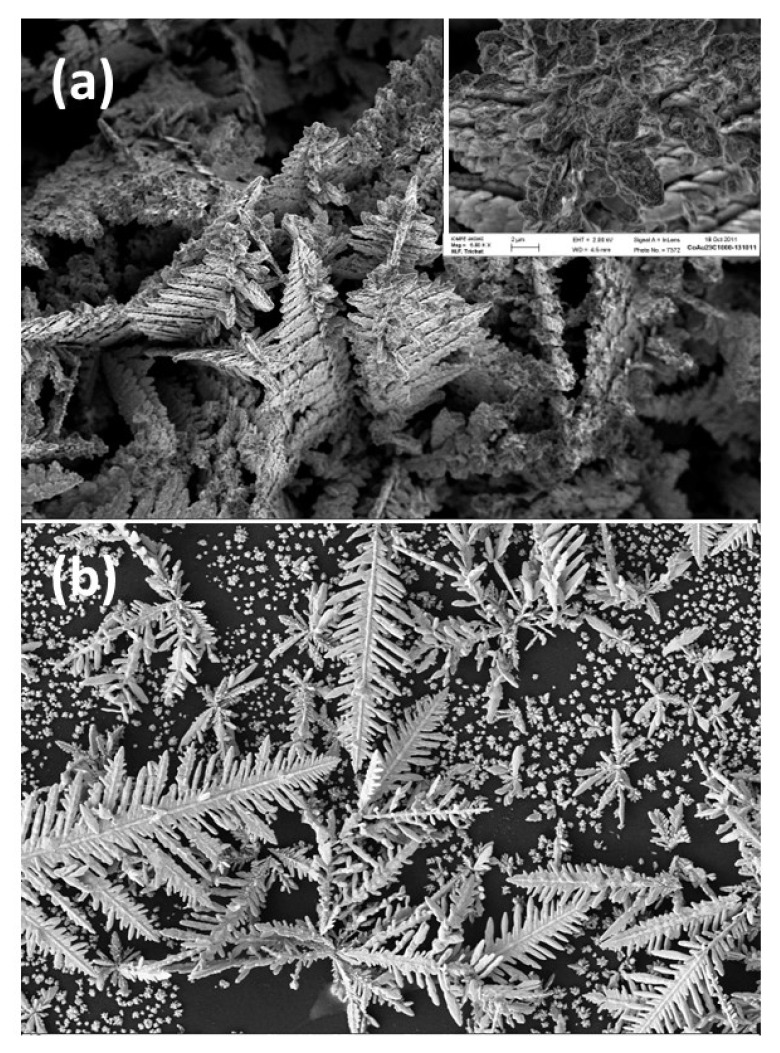
Direct growth of cobalt (**a**) and silver (**b**) dendrites with distinguished multiple levels of branches and sub-branches, using the electrochemical deposition method [34,35]. The insert of (**a**) corresponds to its high magnification. Reproduced with permission from refs. [34,35].

Competition between diffusion and reduction of metal ions leads to the creation of an ion-depleted zone near the surface of the electrode where growth occurs. This results in a higher growth rate at surface prominences, which are more accessible than the surface sites in the roughness cavities of the electrodes. The fluctuation in concentration near the electrode interface leads to the development of a hierarchical structure in terms of multiple levels of branches and sub-branches (Figure 4) [34,35]. During the growth of dendrites, two types of mechanisms can be observed. The first of these mechanisms involves ion-by-ion or atom-by-atom growth and is associated with a range of atomistic phenomena. The second mechanism involves a series of phenomena related to the coalescence of nanoparticles (NPs) and their rotation, as well as interfacial events that lead to total fusion [36,37,38,39].

In contrast to electrochemical deposition, the electrochemical substitution reaction is a non-electrolytic deposition procedure that can generate new nanostructures in a simple, versatile, and powerful way. The difference between the redox potentials of the two involved metals leads to electrochemical substitution processes if the two cations come into contact with each other in the solution phase. Consequently, nanostructures are formed by sacrificing one metal for the other. By controlling the nucleation and growth processes of the nanostructures and tuning the experimental parameters such as metal ion concentration, reaction time, additives, and solvents, it is possible to prepare hierarchical nanostructures with controlled properties [40].

The chemical synthesis is typically conducted in the presence of stabilizers. For instance, in the synthesis of gold (Au) from chloroauric acid solutions, the reduction of Au(III) ions to Au(0) is facilitated by various reducing agents, including citrate, sodium borohydride, and sodium ascorbate. The presence of one or more water-soluble polymers, surfactants, or coating agents has been shown to increase stability and prevent nanoparticle aggregation. Furthermore, the size and morphology of dispersed nanoparticles can be adequately controlled by varying surface modifiers, salt concentration, or reaction conditions. The anisotropic growth of nanoparticles, due to surface modifications, is often the origin of hierarchized structures. Finally, the surface of AuNPs can be readily modified by various biomolecules, including peptides, proteins, antibodies, enzymes, and nucleic acids. A combination of an electrochemical method and surfactant-assisted synthesis was utilized to prepare different hierarchical morphologies of Ag nanoparticles [41].

Chemical Vapor Deposition (CVD) is defined as the chemical reaction of a precursor in the gas phase, resulting in the production of a solid layer on a substrate. The chemical reactions of the precursors occur both in the gas phase and on the substrate. The growth processes can be facilitated or initiated by heat (thermal CVD), high-frequency radiation such as ultraviolet light (photo-assisted CVD), or plasma (plasma-assisted CVD). Furthermore, it has been observed that CVD is increasingly being incorporated into the range of methods employed for the preparation of nanomaterials. Wu et al. [42] reported the one-step growth of metal oxide/carbon nanotube (CNT) sheet composites by water-assisted CVD. The results showed that when the CVD growth process was extended to one hour, CNTs were obtained, and new nanocomposites were grown.

**Figure 5:**
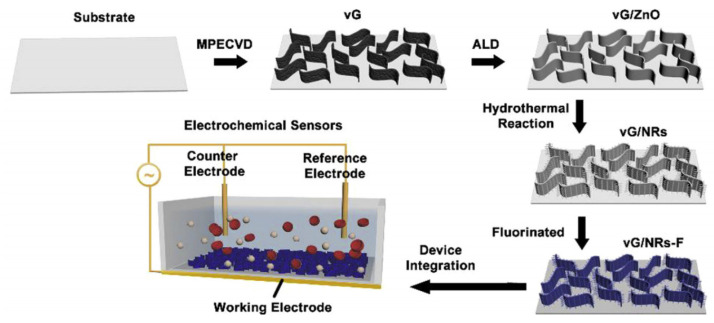
Fabrication process of hierarchical graphene/nanorods structure and the integration of electrochemical sensors based on it [43]. Reproduced with permission from ref. [43].

Consecutive direct growth steps have been utilized to prepare hierarchical structures, including raspberry structures, which consist of the growth of small particles on top of larger particles, or the growth of particles of different morphologies on top of larger unidirectional particles, such as cylinders, nanowires, nanoribbons, and so forth. Hang et al. proposed a self-cleaning and biofouling-resistant electrochemical H_2_O_2_ sensor based on vertical graphene (VG)/nanoparticle stacked construction (Figure 5) [43]. The hierarchical VG/NRs structure consists of branches of ZnO nanorods on vertical graphene nanowalls and was elaborated by combining plasma-assisted chemical vapor deposition, atomic layer deposition, and hydrothermal growth. The fluorinated hierarchical vG/NRs (vG/NRs-F) exhibited favorable liquid repellency and wafer adhesion resistance.

### 2.2. Synthetic Approach Based on Nanomaterials Assembly

**Figure 6:**
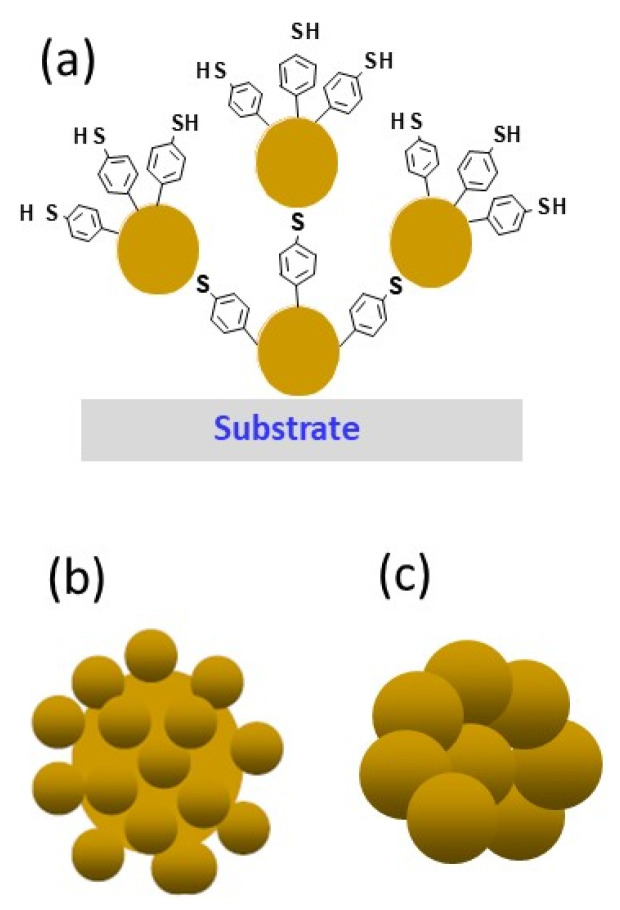
Different designs of assembled nanoparticles: (**a**) Tree particles, (**b**) Raspberry particles, and (**c**) Aggregate particles.

The preparation of hierarchical structures according to this approach occurs through two steps: firstly, the synthesis of nanoparticles using different bottom-up or top-down techniques, and then their assembly to form more complex structures. Porous aggregates based on spherical nanoparticles are one example of such structures, but other assemblies of nanoparticles with different morphologies are also being considered. A variety of strategies have been employed for the assembly of nanoparticles, with the employment of either organic ligands such as diazonium salts or biological molecules like DNA (Figure 6a), or ionic bonds, taking advantage of the nature of the surface charges of the particles. The particles assembled according to the latter strategy can coalesce in a second step (Figure 6b,c).

The agglomeration of nanoparticles is frequently regarded as a consequence of their destabilization, a process that is contingent on their properties (size, shape, surface modification, and concentration) and the solvent in which they are dispersed. In an aqueous solution, the DLVO theory (Derjaguin, Landau, Verwey, and Overbeek) [44] is frequently invoked to elucidate the agglomeration process of nanoparticles. This theory posits that as the size of the nanoparticles increases, their energy barrier and critical concentration threshold concomitantly rise. Conversely, the agglomeration of small nanoparticles is favored by their reduced energy barrier. The DLVO theory has been demonstrated to exhibit a linear relationship between the critical threshold concentration and the particle size at which agglomeration occurs [45,46]. The DLVO theory further posits that the agglomeration of nanoparticles is governed by electrostatic (repulsive) and van der Waals (attractive) interactions [47,48,49]. The strength of these interactions is found to be diminished when electrostatic repulsion predominates over the nanoparticle interactions. Conversely, when electrostatic repulsion is substantially weakened by charge-scavenging effects on the nanoparticle surface, van der Waals interactions become predominant, favoring nanoparticle aggregation. The DLVO theory postulates that nanoparticle agglomeration is promoted by limiting repulsive interactions and/or increasing collision speeds between nanoparticles.

### 2.3. Synthetic Approach Based on Templates

Foam materials are three-dimensional (3D) hierarchical porous skeletons with excellent structural properties, such as a large specific surface area, pore size distribution, and connection. The term “foam” is generally accepted to denote the homogeneous dispersion of gas bubbles in a liquid or a solid [5]. In the case of liquid matrices, the solid foam structure is formed after the liquid has solidified. Metal foams represent a class of solid foams whose matrix is formed from metals or alloys, such as aluminum. The pores, also known as cells, can form either an interconnected network of pores (open-cell foam) or sealed pores (closed-cell foam). Sponge-structured metal foams, with interconnected pores, offer opportunities for solutions to flow through the material and a large surface area for a great interaction between the liquid and the material. This renders them promising candidates for utilization as electrode materials in electrochemical sensors.

A variety of techniques have been developed for the purpose of preparing foam materials. These include liquid melt using a foaming agent [28], gas blowing [5], semisolid stage foaming by compaction of metal powder, and blowing agent [29].

The template method is frequently employed in the fabrication of hierarchical nanomaterials with a foam structure, a process that encompasses several steps. Initially, a template is prepared, which can be a solid matrix, such as alumina, or a nanoparticle-based structure, such as that formed by an assembly of silica nanoparticles, or polymethylmethacrylate (PMMA) latex, or trapped gas and/or chemical reagent in prepared hierarchical materials.

**Figure 7:**
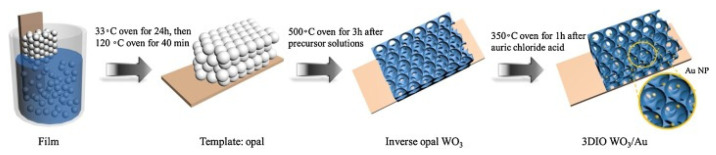
Process diagram for manufacturing 3DIO WO_3_/Au films [51]. Reproduced with permission from ref. [51].

The template method, based on an assembly of nanoparticles, is frequently utilized to prepare nanomaterials with a foam structure. The pre-treated glass substrate is inserted vertically into the PMMA colloidal suspension, thereby obtaining the opal substrate template. The solution is subsequently infiltrated into the opal structure of the substrate, resulting in the formation of a thin film with a porous structure that reproduces the holes between the PMMA spheres. This is achieved by removing them at high temperatures. A further advancement in the field was achieved by Xing et al. [50], who developed a simple sacrificial template method to prepare three-dimensional In_2_O_3_-CuO inverse opal (3DIO) architectures with additional holes. The copper-to-indium molar ratio was found to be a key factor in controlling the number of heterogeneous contacts in In_2_O_3_-CuO composites. Furthermore, Zhang et al. synthesized an acetone sensor based on 3DIO composite material modified with tungsten oxide and Au (WO_3_/Au) by using a sacrificial model method (Figure 7) [51].

In a recent study, Zhao et al. synthesized hierarchical AuNPs@CuO NWs/Cu_2_O/CF nanostructures using a combination of synthesis methods to fabricate wearable electrochemical glucose sensors without enzymes [52]. CuO nanowire/Cu_2_O nanocomposites were synthesized first by in situ growth on a three-dimensional copper foam (CuO NWs/Cu_2_O/CF) and then by electrodeposition of Au nanoparticles (AuNPs@CuO NWs/Cu_2_O/CF). In addition, Lee et al. proposed a facile fabrication of hybrid nanostructures, composed of single-crystal RuO_2_ nanorods on electrospun WO_3_ nanofibres using electrospinning and thermal annealing processes [53], which were used as a catalytic sensing platform for L-ascorbic acid (AA) and hydrogen peroxide (H_2_O_2_) in phosphate-buffered solution (PBS).

In recent years, biomimetic methodologies have facilitated the development of materials exhibiting intricate structures that emulate natural elements, such as those observed on the surfaces of plants and animals [54]. However, reproducing such surfaces is challenging due to their high flexibility and low thermal resistance. In light of these challenges, a range of methods have been devised to reproduce surfaces, with a recent focus on a technique utilizing a magnetic mirror-type magnetron cathode (M3C). This method has been shown to be effective in the sputtering of metals at temperatures below 40 °C while maintaining the integrity of surface patterns inherent in natural organic materials [55,56]. The development of efficient processes for duplicating organic surfaces has allowed the emergence of a range of hierarchical structures.

Recently, biological approaches for the synthesis of nanoparticles have been regarded as being clean, non-toxic, and environmentally acceptable. Various biological agents, including bacteria, actinomycetes, fungi, algae, yeast, and plants, have been utilized [57,58] to prepare nanomaterials. The reduction of metal ions using biological agents can be achieved at ambient temperature and pressure, with a minimal requirement for organic solvents. Furthermore, it has been established that microorganisms, including yeast, bacteria, algae, and fungi, possess the capacity to adsorb and collect metals [59,60]. Furthermore, microbes have been found to release enzymes capable of hydrolyzing metals, thereby facilitating the reduction of metal ions and enhancing the efficiency of the reduction process.

## 3. Performance of Hierarchical Nanostructures-Based Electrochemical Sensors

Hierarchical nanostructures have been shown to increase the surface-to-volume ratio and provide a greater number of active sites, which is advantageous for sensor applications. In addition, the combination of several components in hierarchical nanostructures allows for a synergistic effect to take place, thereby optimizing their sensing performance [17]. The subsequent section will address the performance of electrochemical sensors based on hierarchical nanostructures, with a focus on the synthesis approaches categorized as direct growth, nanomaterial assembly, and template-assisted.

### 3.1. Electrode Materials Prepared with the Direct Growth Approach

Dendritic fractals are a kind of hyperbranched structure that forms at the nanoscale level. Studies on hierarchical fractal patterns in chemical systems have revealed that these structures are potential candidates for the design and production of new electrode materials due to their distinct size, shape, and chemical activity [34]. Furthermore, due to their extremely high surface area and permeability, they exhibit intriguing physical and chemical characteristics. Magnetic iron oxide nanoparticles, for instance, have garnered the interest of researchers due to their excellent biocompatibility, superparamagnetic behavior, and effective interaction with biomolecules. A unique biosensing platform has been constructed based on fractal-patterned iron oxide magnetic nanostructures (FIOMNs) comprising hybrid hemispherical particles [61]. The utilization of hemoglobin (Hb) biosensors in conjunction with screen-printed carbon electrodes (SPCEs) has been demonstrated to facilitate the immobilization process through magnetic means. The resultant biosensors have been shown to exhibit several advantageous characteristics, including high sensitivity, a disposable design, a small sample size, ease of fabrication, and good immunity to interference. This renders them well-suited for H_2_O_2_ screening in real samples. In a related study, Xu’s group described a novel electrode modified with fractal gold (Frac-Au) on an ITO surface, intending to mimic the human nasal membrane [62]. The Frac-Au electrodes exhibited a porous surface that improved the electrochemically active surface and accelerated the electron transfer efficiency when compared to traditional two-dimensional electrodes. Based on Frac-Au nanostructures and enzyme amplification, Liu et al. developed an ultrasensitive sandwich-type electrochemical immunosensor for the quantitative detection of apolipoprotein E4 (APOE4) [63]. The developed APOE4 electrochemical immunosensors demonstrated high specificity, high sensitivity, low detection limit, and a wide linear range.

The prevalence of dendritic silver nanostructures in contemporary research can be attributed to their distinctive electrical, optical, and catalytic properties. Consequently, significant endeavors have been made towards the fabrication of three-dimensional dendritic silver nanostructures. For instance, Wen et al. have synthesized Ag nano-dendrites through a straightforward surfactant-free method, which has been demonstrated to enhance the sensitivity of electrochemical glucose biosensors by one to two orders of magnitude [64]. Hu et al. used electrodeposition on interlaced array microelectrodes (IDAs) to prepare three-dimensional dendritic Ag array (DSA) nanostructures [65]. In addition, this study addressed the production of the 3D dendritic morphology and provided a better understanding of the growth mechanism using electrochemical nucleation theory and nonequilibrium growth kinetics. The performance of all hierarchical sensors with fractal structures is summarised in Table 1.

In a seminal study, Chen et al. [66] pioneered a novel approach to the design of reduced graphene oxide (rGOS) nanocomposites decorated with MnS NPs, utilizing a straightforward ultrasound-assisted method. The sensors fabricated from these nanocomposites exhibited a nanomolar detection limit (3.5 nM) for dopamine, with a linear response within a range of 0.02 to 438.6 µM. In a related study, Naik et al. [67] produced novel ZnS/Au/f-multiwalled carbon nanotubes (MWCNTs) nanostructures using pulsed laser-assisted techniques and wet chemical processes. The ZnS nanospheres were synthesized by pulsed laser ablation of Zn targets in DMSO, which served as both solvent and sulfur source. The electrochemical sensors based on ZnS/Au/f-MWCNT nanocomposites demonstrated the capability for rapid and highly selective detection of a harmful pollutant, namely 4-nitrophenol (4-NP). These sensors exhibited a broad linear dynamic response (10–150 µM), high sensitivity (0.8084 µAµM^−1^ cm^−2^), and a low detection limit using linear scanning voltammetry (30 nM).

In the study by Zhao et al. [68], atomic layer deposition (ALD) was utilized to prepare porous hierarchical iron-based MOF films (PCN-333) on complex planar and 3D substrates that had been precisely fabricated using photolithography. The resultant PCN-333 films exhibited excellent electrochemical activity, which was employed for the detection of dopamine due to their remarkably high sensitivity of 4637.78 µA mM^−1^ cm^−2^, low detection limit, and linearity over a broad range of concentrations.

### 3.2. Electrode Materials Prepared with Nanomaterials Assembly Approach

The assembly of nanomaterials that have been prepared in advance may be a means of fabricating hierarchical nanostructures, with enhanced control over morphology and size. This approach is highly versatile and allows for the combination of nanomaterials that vary in size, morphology, crystal structure, and chemical composition. Recent research has demonstrated that certain core–shell nanostructures, including Ag/WO_3_, Pd/Pt, Au/Pt, Ag/Pt, and Au/Ag, have the potential to function as highly effective catalysts within electrochemical systems.

#### 3.2.1. Nanoparticle/Nanorods

Gold nanoparticles (Au NPs) have recently attracted considerable interest in the domain of electrochemistry, owing to their remarkable properties. These include a high surface area, high chemical stability, superior biocompatibility, high catalytic activity, optical sensitivity, and the ability to enhance electron transport between redox-active biomolecules and electrodes. This property greatly improves the performance of electrochemical sensing [69].

Zinc oxide (ZnO) nanostructures have been utilized as substrates for biosensors, as a consequence of their exceptional properties. These nanostructures possess several advantageous properties that render them suitable for use in immobilizing molecules within biosensors. These properties include high biocompatibility and biomimicry, a high specific surface area, high chemical stability, and a high isoelectric point (IEP 9.5). ZnO nanostructures have also been shown to have excellent potential for efficient carrier transport in redox reactions due to their semiconducting properties [70].

The interaction between the metal substrate and the active catalyst has been shown to affect the electronic structure and surface chemistry of the catalyst, increasing the number of electroactive sites and the charge conductivity [71]. The use of a heterogeneous substrate consisting of a one-dimensional metal and a three-dimensional foam increases the surface area, accelerates electrolyte penetration and ion diffusion, and shortens the charge conduction channels, which contributes to improved kinetics of the detection reaction [72] The combination of noble metals with transition metal supports leads to the formation of a heterogeneous structure that effectively modifies the catalytic activity, selectivity, and stability of the resulting hybrid materials.

**Figure 8:**
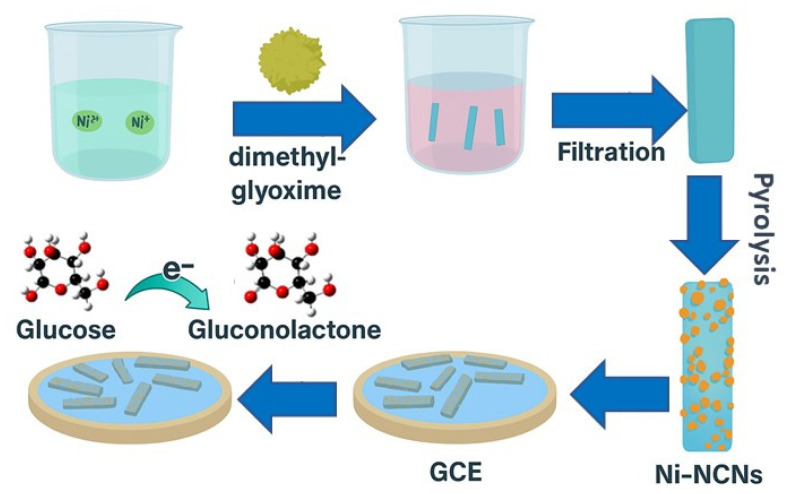
Graphic of the preparation of Ni/NCNs composite for non-enzymatic glucose detection [73].

**Carbon nanomaterials/nanorods:** Due to their high surface area, superior electrical conductivity, and excellent electrocatalytic activity for many redox reactions, carbon nanomaterials such as graphene (Gr), carbon nanotubes (CNTs), and fullerenes have attracted the interest of scientists as promising candidates for electrochemical sensor applications. In addition to the above-mentioned characteristics, Gr also possesses high adsorption capacity and a large surface area. In a study by Hang et al. [43], electrochemical H_2_O_2_ sensors were fabricated based on ZnO nanorods branched on stacked vertical graphene nanowalls (vG/NR) that were fluorinated to possess high liquid repellency and anti-platelet adhesion capabilities. The vG/NRs-F electrode displayed anti-fouling properties after the H_2_O_2_ detection in serum samples. In a separate study, Jia et al. [73] implanted nickel NPs on porous carbon nanorods (Ni/NCNs), which were deposited on glassy carbon electrodes (GCEs) to develop a non-enzymatic glucose sensor, as illustrated in Figure 8. This demonstrated substantial electrochemical activity. The glucose sensors thus fabricated displayed electrocatalytic performance of a remarkable order of magnitude, an ultra-low detection limit, a wide linear detection range, a fast response time (less than 1.6 s), high stability, and anti-interference characteristics.

**ZnO NRs-Au NPs hybrids:** In recent years, significant efforts have been made to fabricate Au-ZnO nanocomposites. For instance, Hou et al. [74] created hybrids of zinc oxide nanorods and gold nanoparticles (ZnO NRs-Au NPs), which exhibited excellent electrocatalytic capabilities for the oxidation of ascorbic acid (AA) and uric acid (UA).

**Figure 9:**
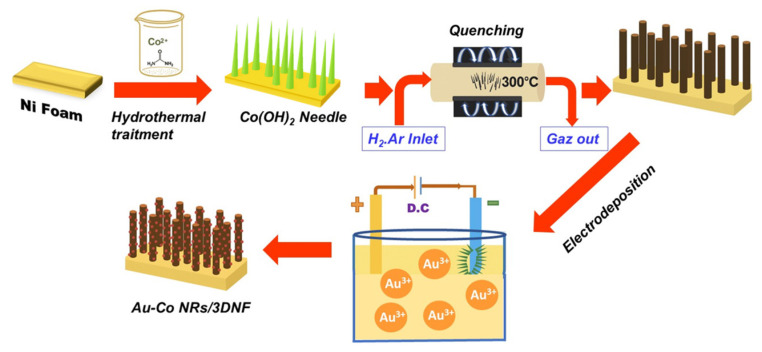
Schematic illustration for fabrication of Au-Co NRs/3DNF hybrid [75].

**Co NRs-Au NPs hybrids:** The decoration of large-area transition metal substrates with gold nanocrystals (Au Ns) has emerged as a potentially effective approach to enhance the catalytic efficiency of resulting materials for sensing applications. In a relevant study, Bach et al. [75] utilized a straightforward synthetic process to synthesize a novel 3D hierarchical nanostructure on a Ni foam substrate (3DNF), the structure being constructed by cobalt nanorods (Co NRs) decorated with Au Ns (Figure 9). The material was then employed as a new self-supporting electrochemical sensor, for which no binder was required for the selective detection of hydrogen peroxide. The excellent sensing performance of the material was attributed to the 3D hierarchical nanostructure, which displayed an architecture that enabled good mass transport and conductivity, and an enhanced number of electroactive sites.

**CuO NRs-Au NPs hybrids:** Au NPs-decorated semiconducting metal oxide nanocomposites have attracted considerable attention in the field of electrochemical sensing applications, owing to their noteworthy electrochemical activity and biocompatibility. In a relevant study, Lei et al. [76] presented a non-enzymatic glucose sensor based on Au/CuO nanosheet composites, which demonstrated a sensitivity of 628.34 µA mM^−1^ cm^−2^ in alkaline environments. Felix et al. [77] described an electrode based on an Au/CuO composite for the non-enzymatic detection of glucose in human urine samples. The sensor displayed a detection limit of 1.4 µM, a reaction time of 3 s, good sensitivity, and long-term stability. Electrodes based on CuO nanorods decorated with Au NPs were reported by Chakraborty et al. [78] and showed a glucose sensitivity of 2009 µAm M^−1^ cm^−2^ and a detection limit of 0.17 µM.

**Figure 10:**
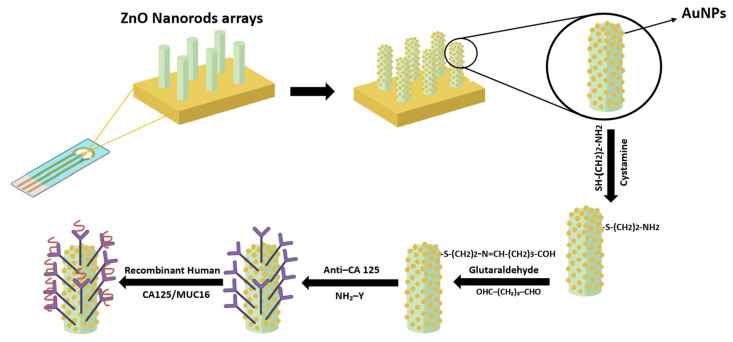
Fabrication schema of ZnO NRs-Au NPs nanohybrids biosensor for CA-125 detection [79].

ZnO NRs-Au NPs hybrids: Gasparotto et al. [79] synthesized nanohybrids made of ZnO nanorods (ZnO NRs) and Au NPs by making them grow on the working electrode to develop a sensor to specifically detect the ovarian cancer antigen CA-125/MUC126, as shown in Figure 10. The incorporation of Au NPs provided a platform conducive to antigen binding, thereby enabling the sensor to demonstrate remarkable performance in the detection of the human ovarian cancer antigen. The sensor exhibited a detection limit of 2.5 ng/μL, which was 100-fold lower than that of the most prevalent immunoblot system.

**MnO_2_ NRs-Au NPs hybrids:** Metal oxide nanocrystals have attracted significant attention in the field of arsenic detection and removal due to their exceptional adsorption capacity and abundant availability. Manganese oxide (MnO_2_) has been extensively utilized in electrochemical sensing due to its cost-effectiveness, high activity in neutral or alkaline media, substantial specific surface area, environmental sustainability, and ease of production. Yang et al. achieved efficient and highly interference-resistant electrochemical detection of As(III) with AuNPs/α-MnO_2_ [80]. The prepared electrodes showed improved reproducibility, a detection limit of 0.019 ppb, and a sensitivity of 16.268 ± 0.242 μA ppb^−1^ cm^−2^, and were applied to detect As(III) in a water sample.

**CuO NRs-Pd NPs hybrids:** high chemical stability and low toxicity. Conversely, noble metals such as Pd, Ag, Pt, and Rh have garnered significant attention due to their exceptional chemical, optical, electrical, and magnetic properties. Palladium has been extensively employed as a noble metal catalyst in oxygen reduction processes, carbon-carbon coupling reactions, methanol, glucose, and ethanol oxidation, and hydrogen evolution reactions. The CuO-Pd nanorods prepared by Chen et al. for glucose detection exhibited a low detection limit, high sensitivity, linearity over a wide concentration range, fast response, high stability, and anti-interference performance [81]. Furthermore, the current response of CuO-Pd nanohybrids was found to be approximately 3.7 times greater than that of CuO and approximately 129.3 times greater than that of Cu(OH)_2_-Pd for glucose detection. The enhanced performance of the CuO-Pd nanohybrids was attributed to a synergistic effect between the CuO and Pd components, indicating a potential for advancement in glucose detection technologies.

**ZnO NRs-Fe_2_O_3_ NPs hybrids:** Given its outstanding electrocatalytic performance, iron oxide has recently become a subject of interest among researchers worldwide as a promising candidate material for sensor fabrication. Its merits include its superior electrocatalytic properties, low cost, ease of fabrication, environmental friendliness, and long-term stability. A highly sensitive non-enzymatic nitrite sensor based on Fe_2_O_3_ nanoparticles encapsulated with ZnO nanorods was fabricated by Ahmad et al. [82]. The sensors demonstrated a high sensitivity (131.2 µA µM^−1^ cm^−2^) and a low detection limit (0.015 × 10^−6^ M).

**Boron-carbon hybrids NRs-Ni NPs:** Following the discovery of carbon nanotubes, considerable interest has been shown in the production and properties of related one-dimensional materials, such as Boron nanotubes and Boron-carbon nanotubes, due to their intriguing electronic properties. Thermal substitution of boron in carbon networks has been demonstrated to enhance their conductivity and electrochemical characteristics. The infusion of nanoparticles into carbon nanomaterials provides them with structural support and improved electrical conductivity. Boron-carbon nanorods adorned with Ni NPs (BC-Ni) have been prepared with high electrochemical capabilities to exploit the unique characteristics of these hybrid nanomaterials for the detection of bacterial pathogenicity. Kaur et al. [83] synthesized highly electroactive BC-Ni nanorods to improve the sensitivity of electrochemical sensors. These nanorods have been shown to selectively detect E. coli O157:H7 with a detection limit of 10 colony-forming units (cfu)/mL and a dynamic detection range of 100–105 cfu/mL.

#### 3.2.2. Nanoparticles/Nanotubes

The surface modification of carbon nanotubes (CNTs) with metals, metal oxides, composite metal oxides, and polymers has been demonstrated to enhance the differential characteristics of CNTs in liquids or to impart new optical, electrical, and magnetic properties.

**MWCNT-metal (Au/Cu/Pt/Pd/Ag) NPs hybrids:** In recent years, there has been a significant increase in the utilization of carbon-based nanostructured materials, including graphene (Gr), multi-walled carbon nanotubes (MWCNTs), and metal nanoparticles, across a wide range of fields and applications. These materials have found diverse applications in areas such as batteries, nanoelectronics devices, and surface-modifying nanomaterials for electrochemical sensors. Carbon nanotubes (CNTs) possess several advantageous properties that make them highly attractive for various applications. These include high conductivity, chemical stability, a large specific surface area, and the ability to reduce surface fouling and accelerate electrochemical processes. A composite of MWCNTs and Au NPs modifying glassy carbon has been reported by Messaoud et al. [84] for the detection of bisphenol A. Under ideal test conditions, the sensor responded linearly to BPA concentrations between 0.01 and 0.7 µM, with a detection limit of 4 nM, one of the lowest obtained to date. Huang et al. utilized composites of MWCNT-Au NPs to produce novel sensitive electrochemical sensors for the selective detection of tyramine, employing a molecularly imprinted polymer (MIP) synthesized using tyramine as a templating agent [85]. Bagheri et al. modified a GCE with Cu NPs decorating reduced graphene oxide-multiwalled carbon nanotube (MWCNT-RGO) nanocomposites, and the electrode exhibited good catalytic activity (pH = 3.0) for the electro-reduction of nitrite and nitrate ions [86]. The sensor was applied for the detection of the analytes in real samples. Eteya et al. fabricated a new electrochemical device for the detection of diclofenac using functionalized multiwall carbon nanotubes (f-MWCNT) with Au-Pt bimetals NPs [87]. Yuan et al. developed electrochemical sensors for nitroaromatic compounds based on three-dimensional porous platinum nanoparticles (Pt-Pd NPs) supported by MWCNTs [88]. The sensors exhibited excellent repeatability, prolonged storage stability, and good anti-interference ability. Furthermore, Enfasi et al. developed an efficient, fast, and stable non-enzymatic glucose sensor by decorating it with Ag NPs and MWCNTs, which had been functionalized with organic molecules [89]. In addition, Bhatka et al. prepared carbon paste electrodes modified with Fe NPs decorated MWCNTs to develop an electrochemical sensor for the detection of uric acid in PBS (pH = 3.0) by differential pulse voltammetry [90]. This method enabled a simple, reliable, fast, reproducible, and inexpensive analysis of UA in biological samples containing ascorbic acid, dopamine, and tyrosine.

**MWCNT-metal oxide (Fe_3_O_4_/ZnO/TiO_2_/CuO/NiO) NPs hybrids:** The use of Fe_3_O_4_ nanoparticles in the construction of sensors and biosensors has attracted increasing interest in academic and industrial circles. The reasons for this interest are severalfold. Firstly, Fe_3_O_4_ NPs exhibit good biocompatibility, interesting superparamagnetic properties, catalytic activity, low toxicity, simple fabrication, and high adsorption capacity. Secondly, they display a high surface area and low mass transfer resistance. The enhancement of their conductive characteristics through synergistic effects can be achieved by producing new hybrid materials by combining CNTs and metal NPs, which have been shown to produce highly sensitive and selective responses to target compounds.

**Figure 11:**
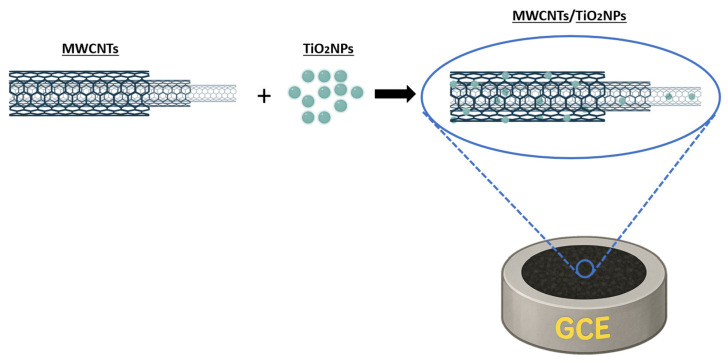
Schematic representation of redox reactions of HQ, CC and RS taking place at the CS-MWCNTs + TiO2NPs/GCE [93].

Madrakian et al. used nanocomposites of Fe_3_O_4_/MWCNT to modify GCEs. The proposed sensors have been effectively used to detect rizatriptan (RZB) in blood samples and real-world drugs [91]. In a separate study, Ghaedi et al. demonstrated that the ZnO-MWCNT/carbon paste electrode (CPE) exhibited outstanding analytical performance for the measurement of citalopram, with extremely low detection limits, high sensitivity, and excellent reproducibility and repeatability when compared to other techniques published in the literature [92]. Fotouhi et al. developed an efficient electrochemical sensor based on MWCNTs and TiO_2_ NPs in a chitosan matrix, as demonstrated in Figure 11. The simultaneous determination of hydroquinone (HQ), catechol (CC), and resorcinol (RS) was achieved with adequate analytical performance in separate or three-component solutions [93]. As demonstrated by Arévalo et al., the utilization of electrochemical sensors based on a GCE modified with MWCNTs/CuO NPs resulted in the quantitative detection of glycerol in biodiesel samples exhibiting good performance, stability, reproducibility, repeatability, low detection limits, and wide linear concentration ranges [94]. Tavana et al. synthesized Pt-Pd/NiO-NPs decorating the surface of single-walled carbon nanotubes (SWCNTs) using a simple chemical precipitation process [95]. The utilization of CPEs based on the nanocomposite material led to the detection of nalbuphine with a detection limit of 0.9 nM and tramadol with a detection limit of 50.0 nM in drug samples. In a related development, Yue et al. proposed electrochemical sensors based on NiO NPs and MWCNTs to detect nitrite [96]. The most advanced device exhibited a sensitive response to the analyte with a linear relationship between the peak oxidation current and concentration ranging from 10^−6^ M to 10^−4^ M (R = 0.997), a sensitivity of 3.53 µA µM^−1^, and a detection limit of 0.25 µM (S/N = 3).

To enhance the optical, magnetic, and electrochemical characteristics of CNTs, the surface of the CNTs was decorated with spinel ferrite nanoparticles of chemical formula MFe_2_O_4_ (M = Mn, Co, Ni, Mg, or Zn). Enfasi et al. produced magnetic nanocomposites of MWCNTs coated with NiFe_2_O_4_ NPs using the sol–gel technique and citric acid [97]. The modified electrodes were then employed for the detection of sotalol in real samples, including drugs, patients, and human urine, due to their exceptional electrocatalytic activity for the oxidation of sotalol at a potential of 500 mV. Utilizing linear scanning voltammetry, the electrode demonstrated linear operation over a substantial concentration range from 0.5 to 1000 μmol L^−1^ of sotalol, exhibiting a detection limit of 0.09 μmol L^−1^.

In addition to carbon nanotubes (CNTs), titanium dioxide nanotubes (TiO_2_NTs) have been identified as a particularly promising solution for immobilizing metal NPs. These nanotubes exhibit high specific surface area, ion exchange capacity, biocompatibility, and photocatalytic properties. According to Chen et al. [98], hybrid nanostructures made of self-assembled TiO_2_NTs, functionalized with an amine moiety, and acting as a support for Au@Pd NPS were used to modify GCEs for the electrocatalytic oxidation of hydrazine, at low potential, with a linear response from 0.06 to 700 µM.

#### 3.2.3. Nanotubes and Nanowires

Nanotubes and nanowires represent a special group of one-dimensional nanostructures that have gained immense attention in electrochemical sensing. Due to high aspect ratio, high electric conductivity, and tailorable surface properties, these nanostructures represent a generalized platform for producing sensors. The recent reports have reflected a special focus on self-assembly of nanowires on electrode surfaces, which leads to percolative and highly conducting networks for optimal electron transfer and large electroactive surface area. Such superior architecture, in association with nanocomposite engineering, results in electrochemical sensors, which are highly sensitive and selective.

Cui et al. prepared a hybrid AuNW-CNT system by non-covalently assembling ultrathin gold nanowires (~1 nm in diameter) inside and outside multi-walled carbon nanotubes (MWCNTs). The composite film was used as an electrochemical and photoacoustic sensor for the detection of α-fetoprotein (AFP) with an ultra-low limit of detection of 0.01 ng/mL. The large surface and conductive nature of MWCNTs, coupled with the plasmonic enhancement of gold nanowires, allowed for effective biomolecule capture and signal amplification. The sensor exhibited good sensitivity, excellent selectivity, and dual-modality performance, and holds great promise for advanced biomedical diagnostics [99].

**Figure 12:**
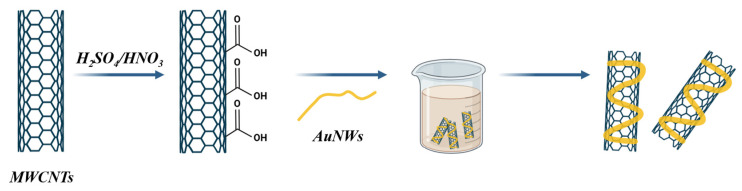
The synthesis of the MWCNTs-AuNWs nanocomposites [100].

Zhang et al. fabricated a hybrid electrochemical sensor based on gold nanowires (AuNWs) combined with multi-walled carbon nanotubes (MWCNTs) (Figure 12). This structure was used to detect pentraxin-3, a cancer biomarker, with a remarkable LOD of 0.16 pg/mL and a sensitivity of approximately 17 µA/(ng·mL^−1^·cm^2^). The AuNW/MWCNT nanocomposite merged the catalytic and conductive properties of gold with the mechanical strength and large surface area of MWCNTs, yielding a highly stable and efficient electrochemical interface [100].

Palve et al. created a bilayer electrode made from copper nanowires (CuNWs) deposited directly onto a glassy carbon substrate and overlaid with a layer of carbon nanotubes (CNTs). The enzyme-free electrochemical glucose sensor had a sensitivity of 1907 μA·mM^−1^·cm^−2^, a limit of detection of 0.33 nM, and a linear range of 10 μM to 2000 μM with a fast response time of under 1 s. The highly electroactive CuNWs combined with conductive, high-surface CNTs allowed for fast glucose oxidation and effective signal transduction. The excellent sensitivity, reproducibility, and fast response of the sensor made it an ideal candidate for point-of-care glucose monitoring without the requirement for an enzyme [101].

As a possible catalyst in the form of alloy structures or hybrid structures with other transition metals, ruthenium dioxide (RuO_2_) has also found common use. Tungsten trioxide (WO_3_) nanostructures have also been studied due to their chemical stability in acidic solutions, robustness, abundance, and optimal electrochemical conductivity. Lee et al. created a heterostructure of RuO_2_ nanorods (NRs) directly grown on electrospun WO_3_ nanofibers (NFs) in a composite form [53]. This structuring greatly enhanced the kinetics of electron transfer, enabling better electrochemical detection of physically relevant molecules like L-ascorbic acid and hydrogen peroxide (H_2_O_2_).

#### 3.2.4. Core-Shell Structures

Core-shell nanocomposites (CSNs) are nanomaterials consisting of an inner layer (core) of one material and an outer layer (shell) of another material. However, recent developments in materials science have led to a slight modification of this definition, allowing it to be applied to a class of nanomaterials in which an outer layer partially or completely covers the inner material. The primary benefit of CSNs lies in their ability to combine the distinct properties of the core and shell, resulting in a composite material that exhibits enhanced or novel physical and chemical characteristics. This is in contrast to the properties of the constituent components. Additionally, the core material is shielded against migration and aggregation, thereby preserving the stability and chemical activity of the nanomaterials over extended periods and spatial dimensions [102].

Core-shell nanostructures, particularly those containing platinum (Pt), have been extensively utilized in the development of electrochemical sensors due to their exceptional catalytic activity. Recent studies have demonstrated that certain core–shell nanostructures, including Ag/WO_3_ [103], Pd/Pt [104], Au/Pt [105], Ag/Pt [106], and Au/Ag [107], function as highly effective catalysts within electrochemical systems. A variety of approaches, including the utilization of carbon-based materials as supports, have been employed to enhance the catalytic activity of these core–shell nanostructures. Graphene, with its substantial surface area and high electrical conductivity, has emerged as a promising platform for the loading of core–shell nanostructures. Khoshroo et al. constructed a sensor (Ag-Pt/Grs/GCE) from Ag-Pt core–shell NPs on graphene nanosheets, which was then used to detect oxazepam in real samples, including serum, urine, and tablets [108]. The sensor’s key advantages are its ease of construction, high specificity and sensitivity, high repeatability, long-term stability, and acceptable accuracy in detecting oxazepam, which could have a wide variety of drug diagnostic applications.

**-Metal-Metal core-shell nanostructures:**

Metal–metal systems with core–shell architectures constitute one of the most established categories of CSNs. Their performance arises from the high electrical conductivity of the metallic cores combined with the tunable catalytic properties of the noble-metal shells. Bimetallic combinations (e.g., Fe@Pt, Au@Pd, Cu@Ag) generate synergistic electronic interactions that enhance charge transfer, increase the density of active sites, and improve structural stability—features that directly benefit electrochemical sensing.

**Figure 13:**
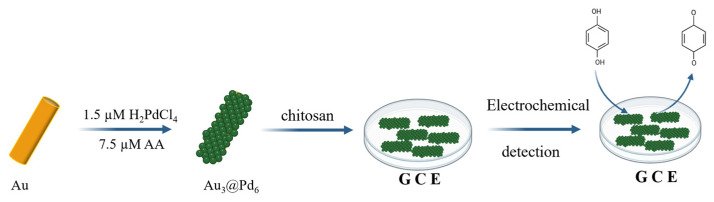
Schematic diagram of the synthetic process of Au3@Pd6 nanocomposite [110].

In a recent study, Mei et al. synthesized Fe@Pt core–shell NPs for the detection of hydrogen peroxide by spontaneous substitution reaction [109]. The amperometric detection of H_2_O_2_ demonstrated linearity across a broad concentration range, spanning from 2.5 μM to 41.605 mM, with a detection limit of 750 nM (S/N = 3) and a sensitivity of 218.97 μA mM^−1^ cm^−2^. In a related finding, Chen et al. described a straightforward and effective methodology for the chemical synthesis of Au@Pd6 NPs (Figure 13) [110]. Electrochemical sensors based on these Au@Pd core–shell nanocomposites exhibited an enhanced capacity to detect hydroquinone (HQ). The experimental findings demonstrated that the GCE modified with Au3@Pd6 nanocomposites (nAu:nPd = 3:6) exhibited electrochemically sensitivity to HQ, with an oxidation peak emerging near 0 V within the potential window spanning from −0.3 to 0.3 V. A novel non-enzymatic immunoassay for ultrasensitive detection of carcinoembryonic antigen (CEA) was developed using β-cyclodextrin-functionalized Cu@Ag shell-core nanoparticles (Cu@Ag-CD) as a label and β-cyclodextrin functionalized graphene nanosheets (CD-GN) as a detection platform [111]. The constructed immunosensors demonstrated exceptional analytical performance for CEA measurement, exhibiting a broad linearity range (0.0001–20 ng/mL), a low detection limit (20 fg/mL), and commendable sensitivity, repeatability, and stability. These findings signify a substantial potential for clinical diagnostic applications.

**-Metal-semiconductor core-shell nanostructures**

Metal–semiconductor architectures combine the plasmonic properties of the metallic core with the redox or photocatalytic activity of the semiconductor shell. Such heterostructures (e.g., Au@Cu_2_O, Au@MnO, Au@CdS) typically form Schottky or p–n junctions that promote charge separation and accelerate interfacial electron transfer. These effects collectively lead to enhanced sensitivities and lower detection limits in electrochemical sensing.

**Au@Cu_2_O(CuO) core-shell nanoparticles:** The advent of nanoscience and nanotechnology has engendered the fabrication of semiconductor-based heterostructures with regulated composition, which can result in technological gadgets with optimal properties. The results of the study demonstrate that hybrid Cu_2_O-based hetero-nanostructures exhibit superior adaptability and distinctive synergistic capabilities due to the integrated interaction of the constituent components, thus indicating potential applications in diverse domains. A particularly noteworthy system is the Au@Cu_2_O heterostructure, which features an Au core and a Cu_2_O shell. The three-dimensional contact between the Au core and the Cu_2_O shell facilitates enhanced metal-shell interaction, thereby promoting plasmonic energy transfer processes and charge transfer between the metal core and the semiconductor shell. In a related study, Su et al. prepared new, highly sensitive non-enzymatic glucose sensors by modifying the surface of GCEs with Au@Cu_2_O nanocomposites. In reference [112], Kumar et al. employed a straightforward seed growth methodology to synthesize Au(core) and CuO(shell) nanoparticles. In the presence of other prevalent vitamins, including riboflavin, ascorbic acid, and uric acid, the modified Au-CuO/MWCNTs/GC electrodes exhibited favorable electrochemical performance for the detection of pyridoxine (PY). Linear calibration curves were obtained for PY concentrations ranging from 0.79 to 18.4 µM, with a detection limit (S/N = 3) of 0.15 µM, and the electrode was successfully employed to determine the content of vitamin B6 in a pharmaceutical tablet sample [113]. Furthermore, these electrochemical sensors demonstrated a linear relationship with glucose concentrations ranging from 0.05 to 2.0 mM under optimal conditions, exhibiting a sensitivity of 715 A mM^−1^ cm^−2^. In a related study, Tang et al. synthesized core–shell NPs consisting of bimetallic AuPd nanocrystals as a core, on which CuO NPs were deposited. The AuPd@CuO NPs were then suspended in distilled water with MWCNTs. This suspension was utilized to modify GCEs, which were employed for glucose determination in basic solution due to their augmented electrocatalytic activity for glucose oxidation [114]. It was demonstrated that at an applied potential as low as +0.34 V, the sensor displayed a good sensitivity of 744.98 µA mM^−1^ cm^−2^ to glucose, a linear concentration range from 3.00 × 10^−5^ to 9.31 × 10^−3^ M, and a LOD of 0.10 µM (S/N = 3). In addition, the modified electrode with AuPd@CuO NPs/MWCNT showed high selectivity and resistance to poisoning by chloride, which makes it suitable for the analysis of glucose in human serum samples.

**Au@SiO_2_ core-shell nanoparticles:** Silicon dioxide is a biocompatible material that facilitates the detection of physiologically active compounds such as dopamine. Its porous structure promotes analyte adsorption and accelerates reaction kinetics when used in composites for sensor fabrication. Gold nanoparticles (AuNPs), with their high specific surface area and excellent electrical conductivity, effectively compensate for the poor conductivity of SiO_2_. In a pioneering study, Yu et al. employed a sol–gel method to synthesize a distinctive core–shell composite consisting of AuNPs and SiO_2_, combined with molecularly imprinted polymers (AuNPs@SiO_2_–MIPs). This composite acted as the molecular recognition element in an electrochemical sensor designed for dopamine (DA) detection [115]. The resulting sensor exhibited high selectivity toward DA over potential interfering species, a low detection limit of 2.0 × 10^−8^ M (S/N = 3), and a wide linear response range from 4.8 × 10^−8^ to 5.0 × 10^−5^ M.

**Au@MnO core-shell nanoparticles:** The low electrical conductivity of materials such as MnO presents a challenge in terms of enhancing the sensitivity of detection; one potential solution is to bind MnO to metal NPs, thereby leveraging the synergistic effects between the two materials to modulate their electronic conduction and catalytic properties. As demonstrated by Zhu et al., the fabrication of Au/MnO core/shell NPs for the electrochemical reduction of H_2_O_2_ resulted in a detection limit of 8 nM [116]. This highly sensitive electrochemical sensor was utilized to ascertain the concentration of H_2_O_2_ emitted from living cells. The results of this study demonstrated that tumourigenic cells released a greater amount of H_2_O_2_ in comparison to non-tumourigenic cells.

**Au@CdS core-shell nanoparticles:** CdS nanoparticles have also been used in the fabrication of biosensor systems, light-emitting diodes, and lasers [117]. Zhang et al. synthesized monodisperse Au@CdS core–shell structures with well-controlled morphology and size by a self-assembly process. The sensors were utilized for the selective catalytic oxidation of dopamine (DA) in the presence of ascorbic acid (AA) and uric acid (UA), exhibiting linearity over a concentration range from 0.002 to 800 μmol L^−1^ with a LOD of 0.55 nM (n = 5, S/N = 3) and high sensitivity. The selectivity in the determination of dopamine was attributable to the electrostatic interactions between the negatively charged Au@CdS core–shell NPs and DA, which was in the cationic form at the determination pH.

**-Metal oxide based core-shell nanostructures:** These systems can be engineered in a wide variety of morphologies, including nanowires, nanorods, nanosheets, nanoporous networks, and more complex hierarchical 3D architectures. Each structural form influences critical parameters such as electron transport, surface accessibility, and catalytic activity, which collectively determine their electrochemical sensing performance, including sensitivity, detection limits, and selectivity.

**TiO_2_ Nanotube:** These have recently attracted considerable attention due to their large surface area, well-aligned nanostructures, high adhesion, and the typical characteristics of TiO_2_, such as high chemical stability, low cost, and good biocompatibility. Guo et al. demonstrated that a bifunctional Ni/CdS Ti@TiO_2_ core–shell nanotube electrode exhibited optimal electrochemical sensing capability [118]. The glucose sensor that demonstrated the highest level of performance exhibited a sensitivity of 1136.67 µA mM^−1^ cm^−2^, a wide concentration range of linearity (between 0.005 and 12 mM), and a low detection limit of 0.35 µM for glucose oxidation.

**Ni/NiO Nanoporous:** Despite the theoretical promise of NiO nanostructures in terms of electrochemical activity, their limited electrical conductivity and electrolyte transport properties present significant challenges when attempting to construct highly performant electrodes. Zhang et al. proposed a Ni/NiO hierarchical porous core structure, in which Ni skeletons with high electrical conductivity are uniformly covered by a continuous thin NiO layer, resulting in highly efficient catalytic action [119]. The porous Ni/NiO multilayer electrode demonstrated efficacy in both the capacity of a pseudo-capacitor, with a surface capacitance of up to 255 mF cm^−2^, and as a sensor due to the electrocatalytic activity for glucose oxidation in 0.1 M NaOH, with a sensitivity of 4.49 mA mM^−1^ cm^−2^ and a detection limit of 10 µM.

**Figure 14:**
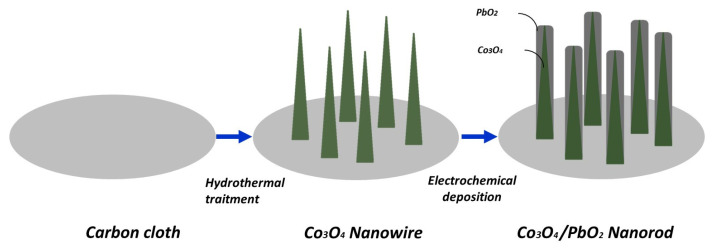
Schematic representation of the synthesis of the Co3O4 nanowire, and the Co3O4/PbO2 nanorod [120].

**Co_3_O_4_/PbO_2_ Nanowire:** To enhance the electrochemical characteristics of Co_3_O_4_, Co_3_O_4_-based core-shell nanostructures with well-defined shapes and customized properties have also been successfully prepared. It has been demonstrated that hybrid nanocomposites allow ions to enter from the shell to the core area or directly touch the collector of the core material. Hybrid metal oxide nanowires with a core–shell structure exhibit superior electrochemical characteristics in comparison to pure Co_3_O_4_ nanoarrays. Chen et al. developed a new electrochemical sensor for glucose sensing that uses Co_3_O_4_/PbO_2_ nano-arrays as electrocatalysts, deposited on a carbon cloth electrode (Figure 14) [120]. The Co_3_O_4_ nanowire array-based multilayer nanocomposite electrode demonstrated optimal sensitivity (460.3 µA mM^−1^ cm^−2^ in the range from 5 µM to 1.2 mM) and a low detection limit (0.31 µM (S/N = 3)).

**Ni_3_S_2_/NiMoO_4_ Nanowires:** Kannan et al. synthesized hierarchical Ni_3_S_2_/NiMoO_4_ nanowires on nickel foam substrates as non-precious metal catalytic electrodes for the electrochemical oxidation of glucose in alkaline solutions [121]. The stacked Ni-Ni_3_S_2_/NiMoO_4_ core–shell nanowires exhibited a superior catalytic response in comparison to their constituent elements, characterized by a rapid reaction time of 1 s, a LOD of 0.055 µM (S/N = 3), and an enhanced sensitivity of 10.49 µA µM^−1^ cm^−2^. These nanowires found application in the determination of glucose levels in serum samples.

**CeO_2_@CuO Core-shell Nanostructures:** Utilizing CeO2@CuO core–shell nanostructures, Dayakar et al. fabricated the fourth-generation glucose biosensors (CeCCS NSs) [122], employing SPEs that were modified with the CeCCS NSs. By chronoamperometry at a potential of +0.4 V, the modified electrode demonstrated a sensitivity of 3319.83 µAm M^−1^ cm^−2^ with a detection limit of 0.019 µM.

**Figure 15:**
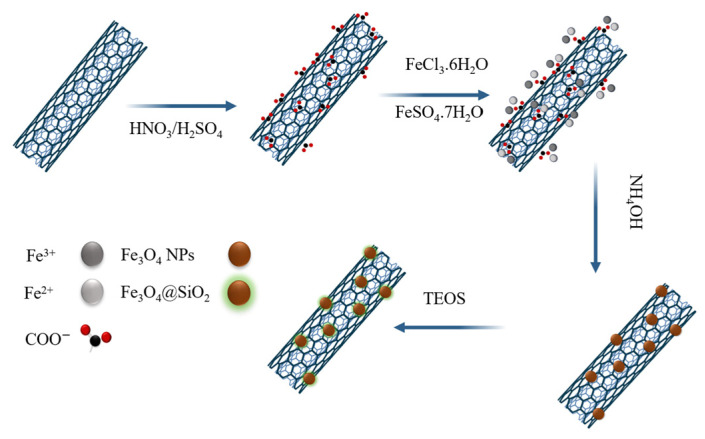
Schematic representation of the preparation process of Fe_3_O_4_@SiO_2_/MWCNT nanocomposite [123].

**Fe_2_O_3_/SiO_2_ Core-shell nanoparticles:** Arvand et al. prepared a new nanocomposite based on MWCNTs decorated with Fe_3_O_4_@SiO_2_ NPs with a magnetic core and used it to fabricate modified carbon paste electrodes (CPEs) [123]. The peak current of the prepared sensor exhibited a linear relationship with the uric acid concentration, ranging from 0.60 to 100.0 µM under optimal conditions, with a detection limit of 0.13 µM. The sensor was employed for the precise measurement of UA in biological fluids (Figure 15).

**-Carbon-based hybrid core-shell nanostructures**

Hybrid systems incorporating carbonaceous shells (graphene, CNTs, amorphous carbon) offer exceptional electrical conductivity, chemical stability, and high surface area. When combined with metal or metal oxide cores, they suppress nanoparticle aggregation, improve electron mobility and significantly enhance sensor performance.

**Au@graphene nanoparticles:** The combination of a metal core and a graphite shell greatly increases the stability of metallic NPs. In addition, this combination gives them some unique characteristics, such as exceptional electrical and catalytic properties. However, under certain conditions, Au NPs have been observed to aggregate and become inactive, a phenomenon that limits their potential applications. The recent creation of metallic nanomaterials separated by graphene shells offers an opportunity to address the aggregation problem of these NPs. Li et al. synthesized Au@graphene core–shell nanoparticles that exhibited high electrical conductivity, catalytic activity, and stability, and the PEDOT/Gr-modified electrode demonstrated high sensitivity for paracetamol detection over a wide concentration range from 0.15 µM to 5.88 mM, with a detection limit of 41 nM (S/N = 3) [124]. In addition, these sensors demonstrated high selectivity and long-term stability, allowing accurate detection of paracetamol in real pharmaceutical samples.

**NiO@graphène core-shell nanoparticles:** NiO nanoparticles exhibit high electrochemical stability and electrocatalytic activity in basic media, attributable to the presence of the Ni^3+^/Ni^2+^ redox couple. They possess a band gap of 3.6 eV as p-type semiconductors. The large potential window and large specific surface area of carbonaceous materials have attracted significant interest from researchers in the fields of electrocatalysis and electrochemistry, resulting in abundant surface-active sites. Consequently, the NiO/C combination can be used to prepare composite electrode active materials, which can exhibit synergistic effects and enhance the overall performance of the electrode. Cui et al. demonstrated significant electrocatalytic activity for glucose oxidation using hierarchical, shell-structured NiO/C microspheres that were synthesized in a controlled manner by simple layer-by-layer hydrothermal assembly [125]. Among the sensors investigated, those with a double-shell structure exhibited the most optimal results, demonstrating linearity over a broader concentration range from 2 µM to 1.279 mM, a remarkable sensitivity of 30.19 mA mM^−1^ cm^−2^, and an extremely low detection limit of 2 µM. The multilayer core–shell architecture of these sensors ensures long-term stability and exceptional resistance to interference.

The performance of all the above-described hierarchical sensors with nanoscale assembly materials is summarized in Table 2.

#### 3.2.5. Sandwich Structure

In the field of electrochemical sensors, the utilization of sandwich structures has emerged as a prominent approach. A notable example can be found in the work of Deng et al., who employed sandwich structures for the fabrication of electrochemical sensing platforms utilizing titanium carbide MXenes. Among the various compounds within this family, Ti_3_C_2_T_x_ has garnered significant attention due to its distinctive lamellar structure and high electrical conductivity, rendering it a promising candidate for the development of electrochemical sensors. However, the major drawbacks are related to the small interplanar spacing of the nanosheets and the low ionic conductivity of pristine Ti_3_C_2_T_x_. The authors attempted to overcome these limits by doping the MXene with poly (3,4-ethylenedioxythiophene): poly (styrene sulphonic acid) (PEDOT: PSS) and RuNPs [126]. First, PEDOT: PSS was inserted into the Ti_3_C_2_T_x_ nanosheets, thus increasing the specific surface area of the composite, and then the surface was decorated with Ru NPs, thereby obtaining the final hierarchical ternary Ru/PEDOT: PSS/Ti_3_C_2_T_x_ nanostructure, which was used to modify GCEs. The material displayed superior electrochemical sensing performance for Sudan I detection, a dye classified as a Class VI carcinogen by the International Agency for Research on Cancer in 2005, exhibiting linearity over a wide concentration range from 0.01 to 100 µM and sensitivity up to 482.43 µA mM^−1^ cm^−2^.

**Figure 16:**
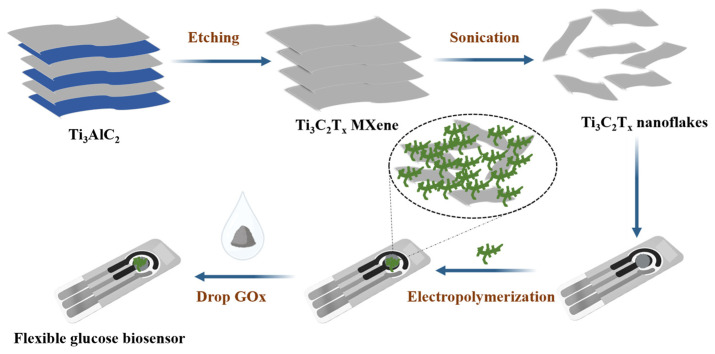
Schematic illustration of the preparation of Ti_3_C_2_T_x_, Ti_3_C_2_T_x_/PANI, and the flexible Ti_3_C_2_T_x_/PANI/GO_x_ flexible glucose biosensor [127].

Wang et al. [127] developed a flexible, wearable biosensing platform based on a hierarchical Ti_3_C_2_T_x_/PANI nanocomposite for non-invasive glucose monitoring in human sweat. The system integrates two-dimensional Ti_3_C_2_Tₓ MXene nanosheets with coral-like polyaniline (Figure 16). This mutual doping effect markedly enhances the composite’s electrical conductivity and specific surface area. The highly interconnected architecture provides a large number of active sites for efficient enzyme immobilization, thereby improving the catalytic efficiency of glucose oxidation. The Ti_3_C_2_T_x_/PANI/GOx-modified electrode fabricated using this approach exhibited excellent analytical performance, including a sensitivity of 25.16 μA mM^−1^ cm^−2^, a detection limit of 26 μM, and a linear range from 0.05 to 1.0 mM. The sensor also demonstrated remarkable mechanical durability, maintaining stable operation under bending angles of up to 60°.

Zhang et al. [128] recently developed a portable biosensing platform based on a highly stretchable three-dimensional Ga@MXene/chitosan (CS) hydrogel network, addressing the challenge of creating skin-friendly sensors that are both flexible and highly conductive. Gallium (Ga) is grafted onto the MXene, enhancing the flexibility and electrical conductivity of the interface. The MXene forms a multidimensional layered conductive matrix, while the CS hydrogel provides superior water absorption, mechanical flexibility, and strong adhesion to the skin—facilitating sweat transport and maintaining reliable contact during movement. The Ga@MXene/CS biosensor demonstrates outstanding glucose detection capabilities, with a low detection limit of 0.77 µM, high sensitivity of 1.122 µA µM^−1^ cm^−2^, and a broad linear range from 10 to 1000 µM, making it well-suited for a variety of physiological monitoring applications. Notably, the device can actively induce perspiration, enabling continuous and reliable sweat-based detection.

**Figure 17:**
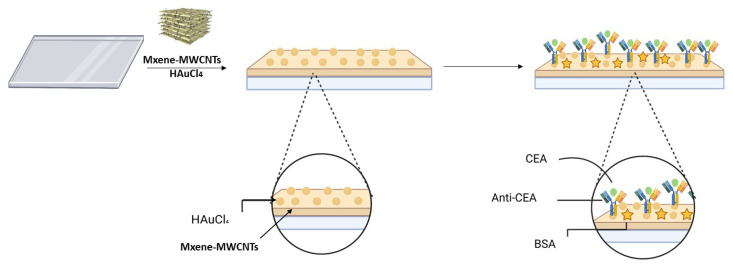
Fabrication Strategy of the MXene–MWCNT Nanocomposite Electrochemical Immunosensor for CEA Detection [129].

Furthermore, Zhang et al. [129] developed an MXene-MWCNT nanocomposite to address the well-known issues of MXene self-stacking and limited electron transport. By integrating this hybrid layer into a BSA/Ab/AuNPs/MXene–MWCNTs–Nafion/ITO configuration (Figure 17), they created a label-free electrochemical immunosensor for CEA detection. The MWCNTs helped maintain separation between MXene sheets, enhanced conductivity at the electrode surface, and increased the number of sites available for immobilizing AuNPs and antibodies. Consequently, the sensor exhibited a wide linear range from 0.050 to 200 ng mL^−1^ and an exceptionally low detection limit of 0.015 ng mL^−1^. Tests on human serum demonstrated recovery rates between 95.34% and 102.09%, with RSDs below 5%, confirming both its accuracy and potential suitability for clinical applications.

### 3.3. Electrode Materials Prepared with Template Approach

Foam materials are 3D porous skeletons with excellent structural properties, such as a large specific surface area and pore size distribution, as well as a good pore connection that allows better ion transport and good stability. These advantages make these materials potential candidates as electrode materials for electrochemical sensor applications. Thanks to the above-mentioned properties, the devices based on foam materials show improved detection sensitivity and good stability as a function of time. Hailin et al. [130] demonstrated that Cu foam-based electrochemical sensors exhibit good selectivity to sucrose. In a recent study, Tao et al. [131] reported on the development of a novel sensor for the detection of glucose based on nickel foam-supported CuO/Co_3_O_4_/r-GO. This sensor demonstrated a high detection performance, with a range of 0.3 to 11.3 mM and a sensitivity of 1000.3 uA mM^−1^. Other groups reported results on AgCu nano-foam prepared by simply dealloying Mg_65_Ag_12.5_Cu_12.5_Y_10_ metallic glass ribbon in citric acid [132]. Electrochemical ethanol sensors based on this material show enhanced performance in terms of a high upper linear limit of 1.5 M and better sensitivity of 109.6 μA mM^−1^ cm^−2^. In comparison with Cu nano-foam, the synergy of these phase structures, with their geometry advantages, gives rise to fast oxidation dynamics, higher electroactive sites, better reversibility, and enhanced microstructure stability. Consequently, this results in a high upper linear limit and an enhanced sensitivity of ∼30 times.

## 4. Conclusions and Future Directions

The utilization of hierarchical nanostructures has become a prevalent approach in the development of electrochemical sensors, a field in which their structural characteristics have been shown to enhance device performance. This review systematically and comprehensively assesses the performance of electrochemical sensors based on various hierarchical structures. In addition, the influence of these nanostructures on selectivity, detection limits, linear range, response time, and other performance metrics has been demonstrated. However, the utilization of diverse hierarchical structures in the fabrication of electrochemical sensors has been accompanied by a paucity of research on the mechanism of action of their structures on electrode performance. A review of the extant literature reveals that the enhanced performance is attributable to synergistic effects and elevated specific surface areas, which facilitate the exposure of additional active sites. In the contemporary context of materials science, the development of approaches for the modulation of nanostructure morphology represents a salient research focus. Consequently, the quest for straightforward and cost-effective methodologies for the synthesis of hierarchical nanomaterials has emerged as a pivotal research priority. Further discussion of this topic will be provided in the subsequent research of our group. Fabricating hierarchical materials necessitates finding an equilibrium between constructing materials with a high specific surface area and limiting the film resistance on the electrode surface. The employment of molecular receptors has been extensively documented as a means to enhance the selectivity of electrochemical sensors; however, this approach concomitantly reduces the number of sites available on the surface of the nanoparticles. In conclusion, to enhance the performance of the sensor, it is essential to consider both the structure of the molecules that will define its surface footprint and the surface topography of the electrode to reduce the shielding effects that also have the consequence of diminishing the active surface.

## Figures and Tables

**Table 1:** Analytical performance of electrochemical sensors based on hierarchical fractal structures.

Analytes	Materials	LODs	Linear Range	Ref.
H_2_O_2_	Fractal iron oxide	0.48 µM	2 to 320 µM	[61]
Thrombin	Fractal Au	5.7 × $10^{-15}$ M	$10^{-15}$ to $10^{-10}$ M	[62]
APOE4	Fractal Au	0.3 ng/mL	1 ng/mL to 10,000 ng/mL	[63]
Nitrate	dendritic Ag	2 uM	0.002–1 mM	[65]

**Table 2:** Electrochemical hierarchical sensors based on nanomaterials assembly.

	Analytes	Materials	LODs	Sensitivity	Linear Range	Ref.
**Biological molecules**	Glucose	Ni NPs-carbon NRs	0.07 µM	210.56 µA ${cm}^{-2}$ ${mM}^{-1}$	0.5336–3.03 mM	[73]
	AA	ZnO NRs-Au NPs	4.699 µM	-	0.1–4 mM	[74]
	UA	ZnO NRs-Au NPs	2.375 µM	-	0.01–0.4 mM	[74]
	H_2_O_2_	Au/Co NRs-3D nickel foam	1.42 µM	-	0.002–0.799 mM	[75]
	glucose	Au NPs-CuO NSs	7.4 µM	628.34 µA ${cm}^{-2}$ ${mM}^{-1}$	-	[76]
	glucose	Au NPs-CuO NRs	1.4 µM	3126.76 µA ${cm}^{-2}$ ${mM}^{-1}$	5 µM to 650 µM	[77]
	glucose	Au NPs- CuO NRs	0.17 µM	1740 µA ${cm}^{-2}$ ${mM}^{-1}$	5 µM to 1.325 mM	[78]
	Glucose	Pd NPs-CuO NRs	<1 µM	2536.9 µA ${cm}^{-2}$ ${mM}^{-1}$	-	[81]
	Tyramine	Au NPs- MWCNT	5.7 × $10^{-8}$ mol/L	-	1.08 × $10^{-7}$ to 1 × $10^{-5}$ mol/L	[85]
	Glucose	Ag NPs-F-MWCNTs	0.03 μM	1057.3 µA ${cm}^{-2}$ ${mM}^{-1}$	1.3 to 1000 mM	[89]
	Uric acid	Fe NPs-MWCNTs	4.80 ± 0.35 × 10^−8^ M	-	7.0 × 10^−8^ to 1.0 × 10^−6^ M	[90]
	Glycerol	CuO NPs- MWCNTs	5.8 × 10^−6^ g dm^−3^	-	9 × 10^−6^ to 1 × 10^−3^ g dm^−3^	[94]
	Glucose	Fe@Pt NPs	750 nM	11.75 µA ${cm}^{-2}$ ${mM}^{-1}$	1–16 mM	[109]
	Glucose	Au@Cu_2_O NPs	18 µM	715 µA ${cm}^{-2}$ ${mM}^{-1}$	0.05–2 mM	[112]
	Vitamin B6	Au@CuO NPs	0.15 µM	-	0.79 µM–18.4 µM	[113]
	Glucose	AuPd@CuO NPs	0.10 µM	744.98 µA ${cm}^{-2}$ ${mM}^{-1}$	3.00 × 10^−5^ to 9.31 × 10^−3^ M	[114]
	Dopamine	Au@SiO_2_ NPs	2 × 10^−8^ M	-	4.8 × 10^−8^–5.0 × 10^−5^ M	[115]
	H_2_O_2_	Au@MnO NPs	8 nM	-	-	[116]
	Uric acid	Au@CdS	0.55 nmol L^−1^	-	0.002–800 mmol L^−1^	[117]
	Glucose	Ti@TiO_2_ NWs	0.35 µM	1136.67 µA ${cm}^{-2}$ ${mM}^{-1}$	0.005–12 mM	[118]
	Glucose	porous Ni@NiO	10 μM	4.49 mA ${cm}^{-2}$ ${mM}^{-1}$	-	[119]
	Glucose	Co_3_O_4_@PbO_2_ NRs	0.31 µM	460.3 µA ${cm}^{-2}$ ${mM}^{-1}$	0.005–1.2 mM	[120]
	Glucose	Ni_3_S_2_@NiMoO_4_ NWs	0.055 µM	10.49 µA ${cm}^{-2}$ ${mM}^{-1}$	0.001–4 mM	[121]
	Glucose	CeO_2_@CuO	0.019 µM	3319.83 µA ${cm}^{-2}$ ${mM}^{-1}$	1 to 8.9 μM	[122]
	Uric acid	Fe_3_O_4_@SiO_2_-MWCNT	0.13 µM	0.03	0.6–100 µM	[123]
	Glucose	NiO@C	2 µM	30.19 mA ${cm}^{-2}$ ${mM}^{-1}$	2 μM–1.279 mM	[125]
**Hazardous Pollutants**	Trace Arsenic(III)	Au NPs-α-MnO_2_ NRs	0.019 ppb	16.268 μA ${ppb}^{-1}$ ${cm}^{-2}$	1 μm–10 mM	[80]
	Nitrite	Fe_2_O_3_ NPs-ZnO NRs	0.015 µM	131.2 µA ${cm}^{-2}$ ${mM}^{-1}$	1 µM to 1250 µM	[82]
	Bisphenol A	Au NPs-MWCNT	4.3 nM	1.76/0.62 µA ${cm}^{-2}$ ${mM}^{-1}$	0.01 µM to 0.7 µM	[84]
	Nitrite+Nitrate	Cu NPs-CNTs	30 nM and 20 nM	-	0.1 to 75 µM	[86]
	Nitro aromatic	Pt/Pd NPs-CNTs	1 ppb	-	3.5 to 190 ppb	[88]
	Benzoate	Fe_3_O_4_ NPs-MWCNTs	0.09 μmol L^−1^	-	0.5–100.0 μmol L^−1^	[91]
	Nitrite	NiO NPs-MWCNTs	0.25 M	3.53 µA ${cm}^{-2}$ ${mM}^{-1}$	10^−6^ M to 10^−4^ M	[96]
	N_2_H_4_	Au/Pd NPs-TiO_2_ NTs	1.2 × 10^−8^ M	-	0.06 to 700 μM	[98]
	Hydroquinone	Au@Pd NPs	0.63 μM	1.127 mA ${cm}^{-2}$ ${mM}^{-1}$	4–5000 µM	[110]
**Disease biomarkers and pathogens**	CA-125	Au NPs-ZnO NRs	2.5 ng/μL	-	-	[79]
	*E. coli*	Ni NPs-BC NRs	10 cfu	-	$10^{0}$ to $10^{5}$ cfu	[83]
	CEA	Cu@Ag NPs	20 fg/mL	-	0.0001–20 ng/mL	[111]
**Pharmaceutical drugs**	Diclofenac	Au Pt NPs-CNTs	0.3 µM	-	0.5 to 1000 μM	[87]
	Citalopram	ZnO NPs-MWCNTs	0.005 μmol L^−1^	-	0.012 to 1.54 μmol L^−1^	[92]
	Dihydroxy-benzène	TiO_2_ NPs-MWCNTs	0.06 μmol dm^−3^	-	0.4–276.0 μmol dm^−3^	[93]
	Sotalol	NiFe_2_O_4_ NPs-MWCNTs	0.09 μmol L^−1^	-	0.5–1000 μmol L^−1^	[97]
	Oxazepam	Ag@Pt NPs-GRs	42 ± 1 nM	0.357 µA ${cm}^{-2}$ ${mM}^{-1}$	0.05–150.0 μM	[108]
	Paracetamol	Au@graphene	0.041 µM	-		[123]

## Data Availability

The original contributions presented in this study are included in the article. Further inquiries can be directed to the corresponding authors.

## Abbreviations

The following abbreviations are used in this manuscript:

LOD	Limit of Detection
NPs	Nanoparticles
NRs	Nanorods
NTs	Nanotubes
NWs	Nanowires
CVD	Chemical Vapor Deposition
CNT	Carbon Nanotubes
DLVO	Derjaguin–Landau–Verwey–Overbeek theory
PMMA	Polymethyl methacrylate
SCPE	Screen-Printed Carbon Electrode
GCE	Glassy Carbon Electrode
MWCNT	Multi-Walled Carbon Nanotubes
CEA	Carcinoembryonic Antigen
PDOT	Poly (3,4-ethylenedioxythiophene)
PSS	Poly (styrene sulfonate)
MOF	Metal–Organic Framework
AA	Ascorbic Acid
PBS	Phosphate-Buffered Saline
